# Motor complications in Parkinson's disease: 13‐year follow‐up of the CamPaIGN cohort

**DOI:** 10.1002/mds.27882

**Published:** 2019-11-11

**Authors:** Han‐Joon Kim, Sarah Mason, Thomas Foltynie, Sophie Winder‐Rhodes, Roger A. Barker, Caroline H. Williams‐Gray

**Affiliations:** ^1^ Department of Neurology and Movement Disorder Center College of Medicine, Seoul National University Seoul Korea; ^2^ John van Geest Centre for Brain Repair, Department of Clinical Neurosciences University of Cambridge Cambridge UK; ^3^ Department of Clinical & Movement Neurosciences UCL Queen Square Institute of Neurology London UK

**Keywords:** levodopa‐induced dyskinesias, motor complications, motor fluctuations, Parkinson's disease

## Abstract

**Background:**

Long‐term population‐representative data on motor fluctuations and levodopa‐induced dyskinesias in Parkinson's disease is lacking.

**Methods:**

The Cambridgeshire Parkinson's Incidence from GP to Neurologist (CamPaIGN) cohort comprises incident PD cases followed for up to 13 years (n = 141). Cumulative incidence of motor fluctuations and levodopa‐induced dyskinesias and risk factors were assessed using Kaplan‐Meyer and Cox regression analyses.

**Results:**

Cumulative incidence of motor fluctuations and levodopa‐induced dyskinesias was 54.3% and 14.5%, respectively, at 5 years and 100% and 55.7%, respectively, at 10 years. Higher baseline UPDRS‐total and *SNCA* rs356219(A) predicted motor fluctuations, whereas higher baseline Mini‐mental State Examination and *GBA* mutations predicted levodopa‐induced dyskinesias. Early levodopa use did not predict motor complications. Both early motor fluctuations and levodopa‐induced dyskinesias predicted reduced mortality in older patients (age at diagnosis >70 years).

**Conclusions:**

Our data support the hypothesis that motor complications are related to the severity of nigrostriatal pathology rather than early levodopa use and indicate that early motor complications do not necessarily confer a negative prognosis. © 2019 The Authors. *Movement Disorders* published by Wiley Periodicals, Inc. on behalf of International Parkinson and Movement Disorder Society.

After a variable period of dopaminergic treatment, Parkinson's disease (PD) patients develop levodopa‐induced motor complications including motor fluctuations (MFs) and levodopa‐induced dyskinesias (LIDs), with important implications for quality of life. Understanding which patients are at high risk for these complications would be of benefit in terms of prognostication and planning management.

The natural history and risk factors for MF and LID are still incompletely understood. Current knowledge is mostly derived from cross‐sectional studies or clinical trials on highly selected patient groups,^1^, ^2^, ^3^, ^4^, ^5^, ^6^ not representative of PD in the population. To overcome this, some studies have employed population‐based PD cohorts,^7^, ^8^, ^9^, ^10^, ^11^ but only 2 have described the incidence and risk factors for MF and LID in a prospective manner, and follow‐up was limited to 5 years.^9^, ^10^

The Cambridgeshire Parkinson's Incidence from GP to Neurologist (CamPaIGN) cohort is an unbiased incident population‐based PD cohort comprising newly diagnosed cases recruited within the county of Cambridgeshire, UK, between December 2000 and December 2002.^12^ This study was established to provide comprehensive and true‐to‐life data on the natural history of idiopathic PD in terms of both motor symptoms and nonmotor symptoms. Baseline data and follow‐up data 3.5, 5, 7, and 10 years from diagnosis have been published previously^12^, ^13^, ^14^, ^15^, ^16^ and shown that there is not only motor but also cognitive heterogeneity in PD and that baseline clinical and genetic variables are predictive of prognosis. Now, follow‐up data to 13 years are available.

Using data from this well‐characterized cohort, we analyzed incidence of MF and LID and their baseline clinical and genetic risk factors, which, to our knowledge, have not been explored in population‐based cohorts followed for more than 10 years. In addition, we examined for the first time the association of MF and LID with later PD outcomes including dementia and death.

## Methods

### Subjects

Subjects were from the CamPaIGN cohort.^12^ Idiopathic PD was diagnosed using UK Parkinson's Disease Society Brain Bank (UKPDSBB) criteria. All cases were followed up at approximately 2‐year intervals. At the 3‐ to 4‐year point, diagnostic reevaluation was undertaken with repeated application of UKPDSBB criteria to maximize diagnostic accuracy.^14^ Of 142 patients included in the last published analysis at 10 year,^16^ 1 was excluded because of a change in diagnosis (essential tremor and osteoarthritis); thus, 141 were included in this study. The study protocol was approved by the local ethics committee, and written informed consent was obtained from all subjects.

### Assessments

At baseline and follow‐up, patients underwent detailed clinical and neuropsychological tests as previously reported.^15^ Disease severity was evaluated using the Unified Parkinson's Disease Rating Scale (UPDRS) and Hoehn and Yahr scale. The presence of MFs and LIDs was assessed using UPDRS section 4. Dementia was diagnosed according to Dianostic and Statistical Manual fourth edition criteria in patients with Mini‐Mental State Examination (MMSE) ≤ 24.

Genotyping was performed for candidate genes with potential prognostic significance including: *MAPT* H1 versus H2 haplotype, *SNCA* rs356219, *APOE* ε‐2/3/4 alleles, *COMT* val(158)met, *BDNF* val(66)met, and *GBA* sequencing for pathogenic variants.^16^, ^17^, ^18^, ^19^

### Statistical Analysis

Time of PD diagnosis was defined as t = 0. Cumulative incidence of MFs and LIDs was calculated using Kaplan‐Meier survival analysis. Time of onset of MFs, LIDs, and dementia was calculated as the midpoint of the interval between the assessment at which the outcome was first recorded and the preceding assessment.

Cox regression analysis was used to investigate covariates that might influence development of MFs and LIDs. All demographic, clinical, and genetic covariates putatively relevant to outcome were evaluated using univariate analyses (Table 1). Multivariate analysis was then performed using covariates with an unadjusted *P* ≤ 0.1 and a backward stepwise approach. Influence of MFs and LIDs on mortality and risk of dementia was analyzed using similar univariate and multivariate Cox regression analyses with MFs and LIDs as time‐dependent covariates. Statistical analyses were performed using IBM SPSS version 22 or SAS.

**Table 1:** Cox regression analysis of predictor variables for development of motor fluctuations and levodopa‐induced dyskinesias

	Motor fluctuations				Levodopa‐induced dyskinesias			
Baseline variables	Unadjusted^d^ HR (95% CI)	*P*	Adjusted^d^ HR (95% CI)	*P*	Unadjusted^d^ HR (95% CI)	*P*	Adjusted^d^ HR (95% CI)	*P*
Sex		0.955				0.149		
Smoking history	0.609 (0.393–0.945)	0.027				0.818		
Years of education		0.397				0.167		
Age at diagnosis		0.548				0.109		
UPDRS3	1.020 (1.001–1.040)	0.043				0.398		
UPDRS total	1.014 (1.000–1.028)	0.054	1.020 (1.005–1.036)	0.010		0.420		
UPDRS dopa‐resistant^a^		0.327				0.755		
HY		0.405				0.939		
MMSE	1.155 (0.986–1.353)	0.074			1.395 (1.095–1.776)	0.007	1.509 (1.170–1.947)	0.002
Tower of London		0.352				0.124		
Pentagon copying		0.308				0.201		
Letter fluency (FAS)		0.254				0.274		
Animal fluency		0.928			1.034 (0.999–1.071)	0.058		
BDI^b^		0.709				0.516		
Motor phenotype (PIGD/intermediate vs tremor‐dominant)	0.586 (0.372–0.924)	0.021				0.691		
LEDD^c^ at baseline	1.001 (1.000–1.003)	0.022			1.002 (1.000–1.004)	0.018		
Levodopa use at baseline		0.113				0.218		
MAPT (H1/H1 vs H2 carrier), n = 130		0.192				1.000		
COMT val158met (Met carrier vs Val/Val), n = 128		0.230				0.166		
BDNF val66met (Met carrier vs Val/Val), n = 128		0.300				0.177		
SNCA rs356219 (A carrier vs GG), n = 124	1.808 (0.982–3.328)	0.057	1.902 (1.034–3.499)	0.039		0.907		
APOE (ε4 noncarrier vs ε4 carrier), n = 124		0.795				0.143		
GBA (mutation carrier vs wild type), n = 113		0.406			2.753 (0.942–8.04)	0.064	4.497 (1.454–13.906)	0.009

a Scores for speech, posture, gait, postural stability, and rising from sitting.

b Beck Depression Inventory.

c Levodopa‐equivalent daily dose.

d ”Unadjusted” indicates univariate analysis, and “adjusted” indicates multivariate analysis.

## Results

Patients were followed up for a maximum of 13 years. Mean follow‐up duration was 7.8 ± 3.5 years. Mean age at diagnosis was 70.2 ± 9.6 years. All patients received dopaminergic therapy during the course of follow‐up. Sixty‐nine were receiving treatment at baseline, of whom 42 were receiving levodopa. No patients had undergone deep brain stimulation or were receiving continuous infusion therapies during the follow‐up period. At 13 years, 61 of 141 patients were alive.

### Risk Factors for MF and LID

**Figure 1:**
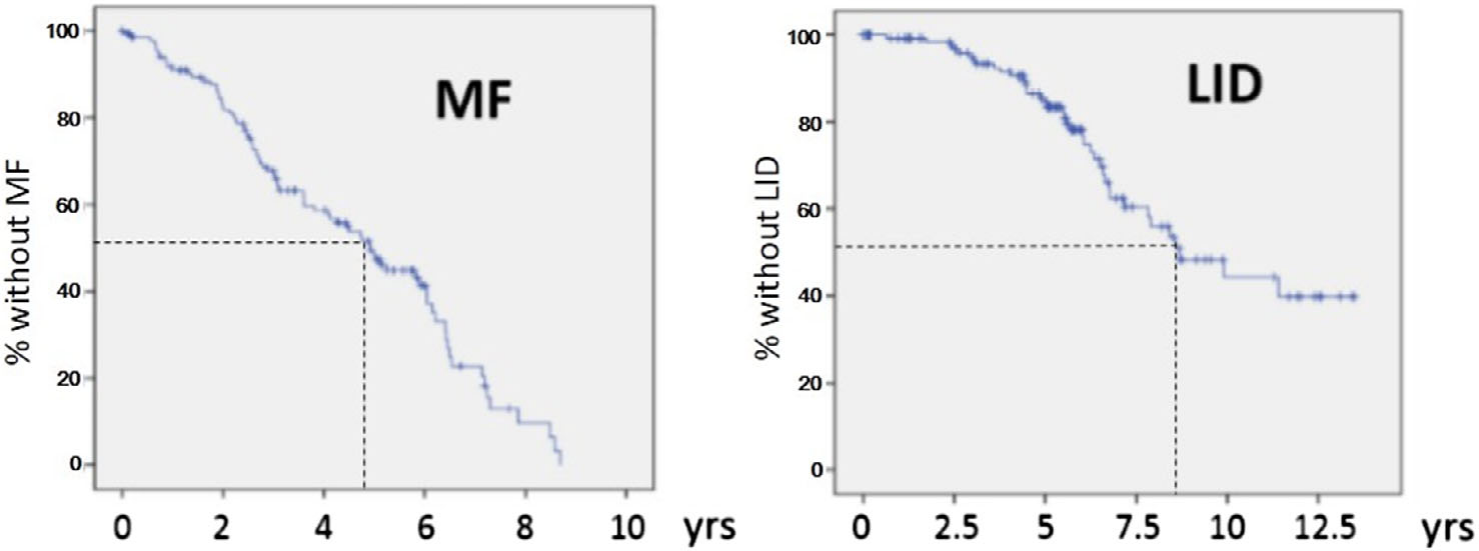
Kaplan‐Meier plots of survival to onset of motor fluctuations (MFs) and levodopa‐induced dyskinesias (LIDs). Dotted line denotes median time to outcome. [Color figure can be viewed at http://wileyonlinelibrary.com]

Eighty‐three patients developed MF, with a cumulative incidence of 54.3% and 100% at 5 and 10 years, respectively. Median time to MF was 4.7 years (Fig. 1). The multivariate model revealed that higher baseline UPDRS‐total and the *SNCA* rs356219 A allele were independently associated with increased risk of MF (Table 1).

LID developed in 39 patients, with a cumulative incidence of 14.5% and 55.7% at 5 and 10 years, respectively. Median time to LID was 8.7 years (Fig. 1). Multivariate analysis showed that higher MMSE at baseline and carrying a *GBA* mutation were independently associated with increased risk of LID (Table 1).

### MF and LID as Prognostic Factors

In univariate analyses, both MF and LID were independently associated with reduced mortality (unadjusted HR, 0.239; 95% CI, 0.133–0.430; *P* < 0.001 for MF; unadjusted HR, 0.355, 95% CI, 0.186–0.676; *P* = 0.002 for LID). Multivariate analysis adjusting for covariates independently associated with mortality (diagnosis age, UPDRS‐total, baseline levodopa use, smoking) showed similar results (adjusted HR, 0.259; 95% CI, 0.136–0.493; *P* < 0.001 for MF; adjusted HR, 0.426; 95% CI, 0.210–0.865; *P* = 0.018 for LID). Stratification by age at diagnosis revealed associations with lower mortality reached significance only in patients over the cohort median of 70 (unadjusted HR, 0.225; 95% CI, 0.114–0.442; *P* < 0.001 for MF; unadjusted HR, 0.296; 95% CI, 0.121–0.724; *P* = 0.008 for LID). Neither MF nor LID was associated with risk of dementia.

## Discussion

This long‐term study in an incident population‐based cohort provides “real‐world” data for the frequency of motor complications in a typical PD population throughout the course of the disease and indicates that at 10 years virtually all surviving patients with PD have developed MF, whereas only 56% experience LID.

Previous population‐based prospective cohort studies have only provided data to 5 years.^9^, ^10^ The cumulative incidence of MF at 5 years was higher, at 54.3%, in our study compared with those studies showing 43% and 23%, which may reflect differences in the population, treatment approaches, and data collection methods. Our high incidence of MF at 5 years appears at odds with the classical view that MF is associated with advanced PD. However, studies have shown that MF can occur as early as 6 months from levodopa initiation,^2^, ^20^ and 1 study showed that 25% of patients initially treated with dopaminergic agonists and 43% of patients initially treated with levodopa developed MF in 2 years.^21^ At 10 years, the cumulative incidence of MF was 100% in our cohort. The Sydney Multicentre Study^22^ similarly reported that all surviving patients had MF at 20 years, supporting our finding that virtually all patients eventually develop MF. An important clinical implication of this is that almost all patients still benefit from dopaminergic treatment in the long term, as evidenced by the presence of MF, and this should be considered when adjusting medications. However, it remains possible that there is a subgroup that does not benefit from levodopa but is censored from analysis at an earlier stage because of death or withdrawal.

It has previously been reported that up to 80% of patients develop LID within 5 years of diagnosis, and 80%–90% of patients experience LID after 10 years,^1^, ^3^, ^23^ but these estimates come from studies of selected cohorts. The older average age of our cohort may be relevant, as patients with a younger age at onset are more likely to develop LID.^10^, ^24^, ^25^ Our 10‐year data are comparable to data from previous retrospective community‐based studies with a similar age at onset,^7^, ^8^ reporting that 53%–59% had LID at 10 or more years. However, the reason for the low cumulative incidence (14.5%) of LID at 5 years, when compared with previously mentioned population‐based prospective cohort studies with a similar age at onset (cumulative incidence at 5 years 24% and 30%^9^, ^10^), is not clear.

In agreement with other studies,^5^, ^9^ our data show MFs are associated with increased baseline disease severity, but in contrast with some previous reports,^2^, ^5^, ^7^ we did not find an association with levodopa use at baseline or levodopa‐equivalent daily dose^26^ in the multivariate analysis. This indicates that early levodopa is not a major factor in driving earlier motor complications, in keeping with studies comparing motor complications in subgroups of patients with differing baseline treatment approaches (levodopa versus dopamine agonists versus no treatment).^27^ This supports the general shift in opinion favoring earlier rather than delayed onset of levodopa treatment.^28^

Higher MMSE at baseline was associated with earlier LID in multivariate analysis, which is a novel finding, but previous studies have not specifically examined the relationship between cognition and LID, to our knowledge. A plausible explanation for this finding is that patients developing early LID represent a subgroup of highly levodopa‐responsive patients in whom pathology is relatively confined to the nigrostriatal system, with minimal extrastriatal and cortical involvement. The observation that LID and MF are not associated with earlier dementia (despite association with disease severity) provides further support for this hypothesis. However, it also is possible that this observation could be influenced by a tendency to use lower medication doses in individuals with cognitive impairment.

Association of the *SNCA* rs356219 A allele and MF is an unexpected finding because this variant has been associated with reduced PD susceptibility and older age at onset,^29^, ^30^, ^31^ as well as reduced plasma α‐synuclein levels in PD.^32^ Our observed association may reflect less severe diffuse α‐synuclein pathology in levodopa‐responsive patients who develop early MF, but it is a finding that requires further replication in an independent cohort.

We observed an increased LID risk in *GBA* mutation carriers, in line with reports that *GBA* mutations are associated with more rapid motor progression,^19^, ^33^ earlier age at onset,^33^, ^34^, ^35^ and possibly earlier DBS surgery.^36^ GBA‐PD is also generally associated with rapid cognitive decline and earlier dementia,^19^, ^33^, ^34^ whereas we found higher baseline MMSE also increased LID risk. However, longitudinal studies have shown that *GBA* status does not determine cognitive status at baseline,^19^, ^34^, ^35^ and we propose that the GBA association with LID is driven by the more aggressive pathology that tends to characterize this condition.

Another novel finding is that MF and LID were associated with reduced mortality in patients >70 at diagnosis, suggesting that levodopa responsiveness predicts better prognosis in older patients. Levodopa‐responsive symptoms are associated with presynaptic nigrostriatal dopaminergic loss,^37^, ^38^ whereas mortality in advanced disease likely results from pathology outside the nigrostriatal system.^16^ This suggests that severity of motor dysfunction at baseline is not necessarily a poor prognostic feature, if the motor deficits are treatment responsive.

In conclusion, our findings are in keeping with the idea that the timing of motor complications reflects the severity of pathology in the dopaminergic nigrostriatal system rather than levodopa use at baseline.^39^ We propose that idiopathic PD patients are on a spectrum from those with disease predominantly confined to the nigrostriatal system, with more prominent motor symptoms at baseline and earlier motor complications, but generally preserved cognition and prolonged survival to those with diffuse α‐synuclein pathology with less treatment responsiveness and less motor complications but more cognitive impairment and reduced survival. Future work is required to develop prognostic models to predict motor complications at the individual patient level.

## Authors’ Roles

Dr. H. J. Kim: drafting the manuscript, study concept and design, analysis and interpretation of data, statistical analysis.

Dr. Sarah Mason: acquisition of data, critical review and revising the manuscript.

Dr. Thomas Foltynie: acquisition of data, critical review and revising the manuscript.

Dr. Sophie Winder‐Rhodes: acquisition of data, critical review and revising the manuscript.

Dr. Roger A. Barker: acquisition of data, critical review and revising the manuscript, study set up and supervision.

Dr. Caroline H. Williams‐Gray: study concept and design, acquisition of data, analysis and interpretation of data, critical review and revising the manuscript, study supervision.

## Financial Disclosures of all authors (for the preceding 12 months)

Dr. H. J. Kim: Employment at Seoul National University Hospital; travel grants from International Parkinson and Movement Disorder Society, Korean Movement Disorder Society; research grants from Seoul National University Hospital, New York University.

Dr. Sarah Mason: employment at Huntington's disease Association and the NIHR Cambridge Biomedical Research Centre awarded to Addenbrooke's Hospital.

Dr. Thomas Foltynie: grant funding from Michael J. Fox Foundation, National Institute of Health Research, John Black Charitable Foundation, Cure Parkinson's Trust and Innovate UK; honoraria for speaking at meetings sponsored by Boston Scientific, Bial, Profile Pharma.

Dr. Sophie Winder‐Rhodes: employment at NHS clinical.

Dr. Roger A Barker: grants from the MRC, Wellcome Trust, Cure Parkinson's Trust, Parkinson's UK, Birax, Huntington's Disease Association and Rosetrees Trust; royalties from Springer Nature and Wiley; consulting fees from UCB, LCT, Oxford Biomedica, Cellular Dynamics International, Cellino, Nova Nordisk, and F‐prime.

Dr. Caroline H. Williams‐Gray: salary funded by an MRC Clinician Scientist Fellowship; additional grant funding from the Rosetrees Trust, the Evelyn Trust, the Michael J. Fox Foundation, Addenbrooke's Charitable Trust, and Parkinson's UK; consultancy work for Modus Outcomes.
